# Inhibitory and Acceleratory Effects of *Inonotus obliquus* on Tyrosinase Activity and Melanin Formation in B16 Melanoma Cells

**DOI:** 10.1155/2014/259836

**Published:** 2014-08-13

**Authors:** Zheng-Fei Yan, Yang Yang, Feng-Hua Tian, Xin-Xin Mao, Yu Li, Chang-Tian Li

**Affiliations:** ^1^College of Chinese Medicinal Materials, Jilin Agricultural University, Changchun 130118, China; ^2^Engineering Research Center of Edible and Medicinal Fungi, Ministry of Education, Jilin Agricultural University, Changchun 130118, China

## Abstract

The aim of the present study is to preliminarily investigate the antimelanogenesis effect of* Inonotus obliquus* extracts by cell-free mushroom tyrosinase assay. It was found that petroleum ether and n-butanol extracts might contain unknown potential tyrosinase inhibitors, while its ethyl acetate extract might contain some unknown accelerators. Six compounds were isolated and their structures were identified by interpretation of NMR data and nicotinic acid was first discovered in* Inonotus obliquus*. In cells testing, betulin and trametenolic acid decreased tyrosinase activity and melanin content, while inotodiol and lanosterol significantly increased tyrosinase activity and melanin content, showing an AC⁡_50_ of 9.74 and 8.43 *μ*M, respectively. Nicotinie acid, 3*β*,22,25-trihydroxy-lanosta-8-ene, had a little or no effect on tyrosinase. Betulin exhibited a mode of noncompetitive inhibition with a *K*_I_ = *K*_IS_ of 0.4 *μ*M on tyrosinase activity showing an IC_50_ of 5.13 *μ*M and being more effective than kojic acid (6.43 *μ*M), and trametenolic acid exhibited a mode of mixed inhibition with a *K*_I_ of 0.9 *μ*M, *K*_IS_ of 0.5 *μ*M, and an IC_50_ of 7.25 *μ*M. We proposed betulin and trametenolic acid as a new candidate of potent tyrosinase inhibitors and inotodiol and lanosterol as accelerators that could be used as therapeutic agent.

## 1. Introduction

Medicinal mushrooms had an established history of being used in nutritionally functional food as well as traditional oriental therapies. Traditional medicines derived from medicinal mushrooms were increasingly being used to treat a wide variety of clinical conditions, with relatively little knowledge of their modes of action. The mushroom* Inonotus obliquus* (Hymenochaetaceae) is a fungus that grew as parasitism on trunks of living brich in the colder northern climates [1–3]. Recently, many reports on* I. obliquus* had been published concerning the health-promoting effects, including anticancer effects, immune-stimulating activity [4–8]. More studies focused on the antioxidant capacity and structure activity studies of components of* I. obliquus* [9]. However, up to now, the little efforts have been addressed to screen tyrosinase inhibitors from* I. obliquus*. The color of skin is determined by melanin [10]. The major role of melanin is to protect the skin from damaging effects of ultraviolet radiation [11]. Melanin biosynthesis is a well-known physiological response of human skin upon exposure to ultraviolet light and other stimuli. Melanogenesis is regulated by enzymes such as tyrosinase [12]. Tyrosinase plays a crucial role in the initial step of melanin synthesis by catalyzing the oxidation of L-tyrosine (L-Tyr) to 3,4-dihydroxyphenylalanine (DOPA) and the oxidation of DOPA to dopaquinone [9, 13–15]. Oxidative polymerization of several dopaquinone derivatives gives rise to melanin. Nowadays, increasing the awareness of skin-whitening, demand for whitening products was progressively increased; tyrosinase inhibitors had become a hot spot on research of whitening additive. A study was undertaken to investigate if* I. obliquus* have any antimelanogenesis effects with a view of its possible use as a treatment for hyperpigmentation and a skin-whitening agent in cosmetics.

In this report, we describe the differential extraction of dried and powdered* I. obliquus* with solvents of different polarity. The ability of the different extracts to act as a skin-whitening agent was evaluated by its ability to inhibit tyrosinase, the rate limiting enzyme in melanogenesis. Initially, a cell-free mushroom tyrosinase system has commonly been employed for the testing and screening of potential skin-whitening agents [16]. We sought to isolate the active compounds from* I. obliquus* extracts used as tyrosinase inhibitors. A bioassay against mushroom tyrosinase was used to identify potential compounds. Then potential components were tested for cellular antityrosinase activity and kinetically analyzed in B16 melanoma cells. Kojic acid, that is well known to be an inhibitor of tyrosinase and melanogenesis, was used as a positive control [17].

## 2. Materials and Methods

### 2.1. Reagents

Mushroom tyrosinase (EC1.14.18.1), Dimethyl sulfoxide (DMSO), L-tyrosine (L-Tyr), L-3, 4-dihydroxyphenylalanine (L-DOPA), and *α*-melanocyte stimulating hormone (*α*-MSH) were purchased from Sigma (St. Louis, MO, USA). All other reagents were of analytical grade. The water used was redistilled and ion-free.

### 2.2. Preparation of Samples

Powdered* Inonotus obliquus* (120 g) purchased from Nanjing Mushroom Biotechnology Co., Ltd, was extracted for 15 mins three times with petroleum ether using a reflux apparatus. The extracts were filtered and the filtrate was collected and then freeze-dried (F1, 0.2 g). The solid residues were extracted with ethyl acetate; the filtrate was collected and then freeze-dried (F2, 0.3 g). In turn, the formed residues were extracted with n-butanol and water. The two collected filtrate was collected and freeze-dried, respectively (F3 (n-butanol fraction), 0.6 g; F4 (aqueous fraction), 0.4 g resp.).

### 2.3. Isolation of Tyrosinase Inhibitory Compounds

The Shimadzu LC-20AT series high performance liquid chromatography system was equipped with a diode array detector (DAD). Analysis was carried out using an Inertsil ODS-SP column (250 mm × 4.6 mm i.d., 5 *μ*m). A linear gradient elution of eluents A (methanol) and B (water) was used for separation. The elution program was optimized and conducted as follows: a linear gradient of 5% B (0–10 min) and 3% B (11–50 min). The peaks were recorded using DAD absorbance at 205 nm and the solvent flow rate was 0.5 mL/min and the oven temperature was set at 25°C. The samples were first dissolved in methanol. The solutions (2 mL) were filtered through a 0.45 *μ*m membrane filter prior to HPLC analysis. The injection volume for samples was 10 *μ*L. The preparative high performance liquid chromatography (PHPLC) was equipped with a semipreparative column. The chromatographic system consisted of a Shimadzu binary pump and Shimadzu SPD-20A photodiode array detector (PDA). A semipreparative column (Shima-Packed Column (250 mm × 10 mm), PREP-ODS) was used for separation. Mobile phase was methanol (A)-water (B). The flow rate was 1 mL/min and PDA was performed 205 nm. The gradient separation was programmed as the following: mobile phase B was started with 5% in 10 min, to 3% in next 40 min till the separation programme ended. Four fractions (F1-F4) were purified to Fa, Fb, Fc, Fd, respectively. New formed fractions (Fa-Fd) were redissolved with DMSO to a proper concentration for cell-free mushroom tyrosinase assay, and then compounds were obtained by PHPLC for the enzymatic assay in B16 melanoma cells.

### 2.4. Determination of Tyrosinase Activity in Fractions

Enzymatic assay was performed for screening active fractions according to the procedure of Chen and Liu [18, 19]. The test fractions or kojic acid were first dissolved in DMSO at 10 *μ*g/mL. Mushroom tyrosinase and L-Tyr were reconstituted in 50 mM Na_2_HPO_4_-NaH_2_PO_4_ buffer (pH 6.8) at 1000 U/mL and 2.5 mM, respectively. A mixture of 60 *μ*L of 50 mM Na_2_HPO_4_-NaH_2_PO_4_ buffer (pH 6.8) and 100 *μ*L L-Tyr was designated as solution 1 (control group). The solution 2 consisted of 20 *μ*L of 50 mM Na_2_HPO_4_-NaH_2_PO_4_ buffer (pH 6.8), 40 *μ*L compounds or kojic acid, and 100 *μ*L L-Tyr. Solutions 1 and 2 were added to 40 *μ*L tyrosinase for 6 min reaction time at 37°C, individually. Absorbance of the resulting solutions was recorded every min by Beckman TU-1810 spectrophotometer at 475 nm. One unit of tyrosinase activity was arbitrarily defined as a rate of increase of 1 absorbance unit per min in the initial linear region of a plot of absorbance against time. The tyrosinase activity determined by the increasing absorbance at 475 nm accompanying by the oxidation of the substrates was calculated as the following formula [20]:

(1) $$\begin{array}{c} \text{Tyrosinase}\;\text{activity}\;\%=\frac{A2}{A1}\times 100, \end{array}$$

where *A*1 is absorbance at 475 nm with solutions 1 and tyrosinase and *A*2 is absorbance at 475 nm with solutions 2 and tyrosinase.

For active fractions that inhibited mushroom tyrosinase by above method described at 0, 4, 6, 8, 10, and 12 *μ*g/mL, the extent of inhibition or acceleration was here expressed as the concentration of samples needed to inhibit or accelerate 50% of enzyme activity (IC_50_, AC_50_) [21], and then they investigated the effects on cellular tyrosinase activity, melanin content, and cytotoxicity test of B16 melanoma cells.

### 2.5. Cell Culture and Treatment

The B16 melanoma cells were purchased from the Type Culture Collection of the Chinese Academy of Sciences (Shanghai, China). The cells were cultured in Hyclone's Modified RPMI-1640's Medium (Hyclone, Thermo Fisher Scientific, USA) containing 10% fetal bovine serum, 1% Penicillin-Streptomycin Solution, and 100x (Beyotime Institute of Biotechnology, China) in culture flasks in a CO_2_ incubator with a humidified atmosphere containing 5% CO_2_ in air at 37°C. The cell culture medium was changed every 2-3 days and subcultured by trypsinisation after beginning to adhere and grow for 3 days. The cells were seeded at the appropriate cell density by using BD Accuri C6 (BD, USA) into wells of cell culture plates for further experiments.

### 2.6. Cell Viability and Apoptosis Rate of Compounds

To determine the safety of the various compounds, after treatment with the test compounds cell viability was determined by using MTT colorimetric assay and cell apoptosis rate by using AnnexinV-FITC apoptosis analysis kit (Tianjin Sungene Biotech Co., Ltd). 1 × 10^6^ cells were added to individual wells of a 24-well plate. After 24 h incubation, test compounds or kojic acid (100, 200, 400 *μ*M) were added to each well and incubated for another 72 h. Cell viability was determined in a colorimetric assay using mitochondrial dehydrogenase activity in active mitochondria to form purple formazan. Apoptosis rate in a fluorochrome assay using flow cytometry (BD, USA).

### 2.7. Determination of Cellular Tyrosinase Activity and Melanin Content in Compounds

Cellular tyrosinase activity and melanin content were measured using a previously described method [22] with small modifications. The B16 melanoma cells were seeded with 1 × 10^6^ cells/well in 3 mL of medium in 6-well culture plates and incubated overnight to allow cells to adhere. The cells were exposed to various concentrations (100, 200, and 400 *μ*M) of compounds or kojic acid for 72 h in the presence or absence of 100 *μ*M *α*-MSH (at 0 *μ*M as control group). For cellular tyrosinase activity, the cells were washed with PBS and lysed with PBS (pH 6.8) containing 1% Triton X-100. Then, the cells were disrupted by M-PER mammalian protein extraction reagent (Pierce, Rockford, IL, USA), and the lysates were clarified by centrifugation at 10,000 ×g for 10 min. Protein content was determined using a commercial protein assay kit (Bio-Rad, Hercules, CA). After quantifying protein levels, the protein concentration was adjusted with lysis buffer until each lysate contained the same amount of protein (40 *μ*g). A mixture of 40 *μ*L of 0.1 M PBS buffer (pH 6.8) and 100 *μ*L of L-tyrosine was designated as solution 3. The solution 4 consisted of 20 *μ*L of 0.1 M PBS buffer (pH 6.8), 20 *μ*L of various concentrations of test compounds or kojic acid, and 100 *μ*L of 2.5 mM L-DOPA dissolved with 0.1 M PBS buffer (pH 6.8). Solutions 3 and 4 were added to 40 *μ*g protein for 30 min reaction time at 37°C, individually. After incubation at 37°C, the absorbance was measured at 475 nm. Tyrosinase activity in the protein was calculated by the following formula [23]:

(2) $$\begin{array}{c} \text{Tyrosinase}\;\text{activity}\;\%=\frac{A3}{A4}\times 100, \end{array}$$

where *A*3:  absorbance at 475 nm with Solutions 3 and protein; *A*4: absorbance at 475 nm with Solutions 4 and protein.

For melanin content, the cells were treated with test compounds or kojic acid in the presence or absence of *α*-MSH as described above. The cells were washed with PBS and lyzed with 800 *μ*L of 1 N NaOH (Sigma, USA) containing 10% DMSO for 1 h at 80°C. The absorbance at 400 nm was measured. The melanin content was determined from a standard curve prepared from an authentic standard of synthetic melanin (Sigma, USA).

### 2.8. Kinetic Analysis of Tyrosinase Activity Inhibition Analysis by Compounds

The cells were treated with test compounds as described above for the determination of tyrosinase activity. Each well of a 96-well plate contained 40 *μ*g of lysate protein, 20 *μ*L of 0.1 M PBS buffer (pH 6.8), 20 *μ*L of various concentrations of test compounds or kojic acid at 0, 50, 100, 200, and 300 *μ*M (at 0 *μ*M as control group), and 100 *μ*L of various concentrations L-DOPA (0.125, 0.25, 0.5, 1, and 2 mM) as substrate. After incubation at 37°C for 30 min, the absorbance was measured at 475 nm. The inhibition constants for compounds and inhibition type were calculated using Lineweaver-Burk plot. The Lineweaver-Burk plot was widely used to determine important terms in enzyme kinetics, such as *K*_m_ and *V*_max⁡_. The plot provided a useful graphical method for analysis of the Michaelis-Menten equation: *V* = *V*_max⁡_  [*S*]/(*K*_m_ + [*S*]). It took the reciprocal gave Lineweaver-Burk plot: 1/*V* = (*K*_m_ + [*S*])/*V*_max⁡_  [*S*] = ((*K*_m_/*V*_max⁡_)(1/  [*S*])) + (1/*V*_max⁡_), where *V* is the reaction velocity (the reaction rate), *K*_m_ is the Michaelis-Menten constant, *V*_max⁡_ is the maximum reaction velocity, and [*S*] is the substrate concentration. The y-intercept of such a graph was equivalent to the inverse of *V*_max⁡_; the x-intercept of the graph represents −1/*K*_m_. It also gave a quick, visual impression of the different forms of enzyme inhibition. The inhibition constant (*K*_I_ or *K*_*IS*_) was generated from the slope of the apparent *K*_max⁡_/*V*_max⁡_ or  1/*V*_max⁡_ versus the concentrations of compounds.

## 3. Results 

### 3.1. Determination of Tyrosinase Activity in Fractions from* Inonotus obliquus*

**Figure 1:**
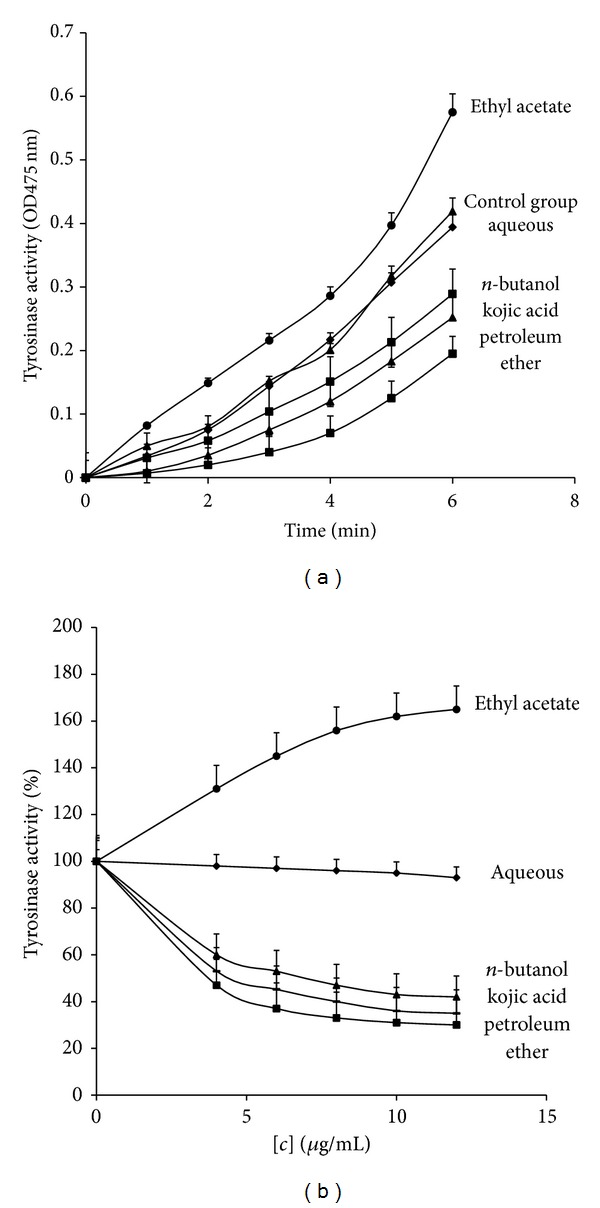
Screening of tyrosinase inhibitors with using Tyr as the substrate, concentrations of fraction were 10 *μ*g/mL (a); inhibition (acceleration) 50% of enzyme activity (IC_50_, AC_50_) was determinated by tyrosinase activity versus the concentrations of every fraction (0, 4, 6, 8, 10, 12 *μ*g/mL) (b).

Due to the colour interference of the extract, the tyrosinase inhibitory effect of original extracts from* I. obliquus *(F1-4) was unable to be determined. Therefore, the extract of* I. obliquus* was first separated and collected as fractions a-d on a PHPLC. Each fraction was subjected to cell-free mushroom tyrosinase assay of tyrosinase inhibitory activity. The result was shown in Figure 1, petroleum ether (Fa) and n-butanol (Fc) fractions showed tyrosinase inhibitory activity (IC_50_ = 3.81, 6.32 *μ*g/mL, resp.). Petroleum ether fraction (Fa) had stronger inhibitory effect than kojic acid (IC_50_ = 5.23 *μ*g/mL). On the contrary, ethyl acetate (Fb) fraction had acceleration effect (AC_50_ = 7.12 *μ*g/mL). It was suggested that (i) the mushroom tyrosinase assay was a rapid assay for the screening of potential skin-whitening agents. (ii) There were both inhibitors and accelerators in* I. obliquus* by extrapolation. (iii) The aqueous fraction (Fd) did not show any effect. This could be due to a polar agent present in aqueous fraction that was different from the nonpolar agent seen in petroleum ether fraction. So the inhibitory effect was small and it was not economically feasible to be developed further.

### 3.2. Analysis and Identification of Compounds in Fractions from* I. obliquus*

**Figure 2:**
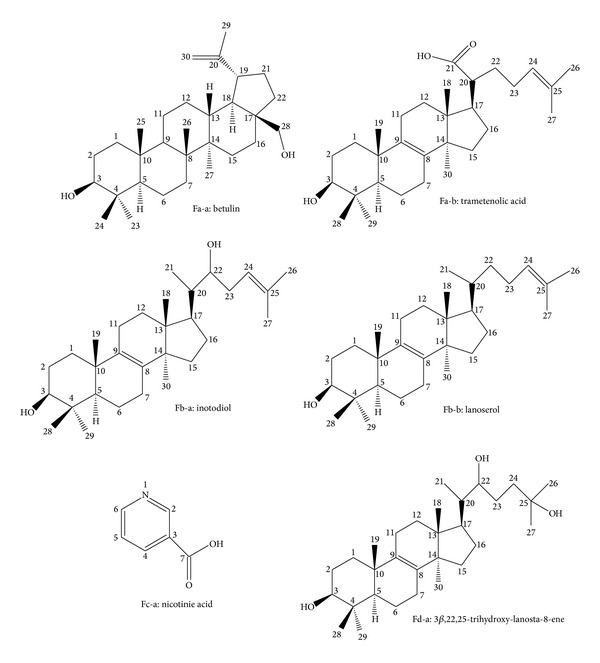
Chemical structure of betulin (Fa-a), trametenolic acid (Fa-b), inotodiol (Fb-a), lanoserol (Fb-b), nicotinie acid (Fc-a), and 3*β*,22,25-trihydroxy-lanosta-8-ene (Fd-a).

The number of compounds in fraction a-d (Fa-d) was analyzed by HPLC, respectively. In the HPLC chromatogram, there were two, two, one, one major peaks (Fa-a, Fa-b, Fb-a, Fb-b, Fc-a, Fd-a) in different fractions. Compound Fa-a, Fa-b, Fb-a, Fb-b, Fc-a, Fd-a were further obtained by PHPLC. The structural information of six compounds was obtained using NMR. All spectral data were consistent with the data of known betulin (Fa-a), trametenolic acid (Fa-b), inotodiol (Fb-a), lanoserol (Fb-b) nicotinie acid (Fc-a), and 3*β*,22,25-trihydroxy-lanosta-8-ene (Fd-a) (Figure 2) [24–27].

*Fa-a*. Betulin, ^1^H-NMR (300 MHZ, CDCL_3_): 1.01 (s, 3H, 23-Me), 0.83 (s, 3H, 24-Me), 0.97 (s, 3H, 25-Me), 0.95 (s, 3H, 26-Me), 0.89 (s, 3H, 27-Me), 3.30,3.26 (dd, 1H, J = 10.8,3-CHOH), 3.77,3.72 (dd, 1H, J = 10.7,28-CHOH), 4.60 (d, 2H, J = 5.2,20-CH2). ^13^C-NMR: see Table 1.

*Fa-b*. Trametenolic acid, ^1^H-NMR (300 MHZ, CDCL_3_): 0.76 (s, 3H, 18-Me), 0.97 (s, 3H, 19-Me), 1.59 (s, 3H, 26-Me), 1.68 (s, 3H, 27-Me), 0.99 (s, 3H, 28-Me), 0.80 (s, 3H, 29-Me), 0.89 (s, 3H, 30-Me), 1.04 (t, 1H, 5-H), 1.40 (m, 1H, 17-H), 3.21,3.19 (dd, J = 130.0,1H, 3-CHOH), 5.19 (t, 1H, 24-H). ^13^C-NMR: see Table 1.

*Fb-a*. Inotodiol, ^1^H-NMR (300 MHZ, CDCL_3_): 0.73 (s, 3H, 18-Me), 0.99 (s, 3H, 19-Me), 0.94 (d, 3H, 21-Me), 1.57 (s, 3H, 26-Me), 1.66 (s, 3H, 27-Me), 0.98 (s, 3H, 28-Me), 0.81 (s, 3H, 29-Me), 0.88 (s, 3H, 30-Me), 1.05 (t, 1H, 5-H), 1.57 (m, 1H, 17-H), 1.80 (d, 1H, 20-H), 3.24,3.21 (dd, J = 4.41H, 3-CHOH), 3.6,3.46 (m, J = 11.4,1H, 22-CHOH), 5.19 (t, 1  H, 24-H). ^13^C-NMR: see Table 1.

*Fb-b*. Lanoserol, 1H-NMR (300 MHz, CDCL_3_): 0.691 (s, 3H, 18-Me), 0.812 (s, 3H, 29-Me), 0.876 (s, 3H, 30-Me), 0.913 (d, 3H, 21-Me), 0.980 (s, 3H, 19-Me), 1.001 (s, 3H, 28-Me), 1.050 (m, 1H, 5-H), 1.400 (m, 1H, 20-H), 1.480 (m, 1H, 17-H), 1.603 (s, 3H, 27-Me), 1.683 (s, 3H, 26-Me), 3.22 (dd, J = 4.43,1H, 3-CHOH), 5.10 (t, 1H, 24-H). ^13^C-NMR: see Table 1.

*Fc-a*. Nicotinie acid, 1H-NMR (400 MHz, CDCL_3_)13.45 (s, 1H, 7-COOH), 9.07 (s, 1H, 2-H), 8.79 (s, 1H, 4-H), 8.28 (d, 1H, 5-H), 7.54 (d, 1H, 6-H).

*Fd-a*. 3*β*,22,25-Trihydroxy-lanosta-8-ene, 1H-NMR (300 MHZ, CDCL_3_): 0.92 (s, 3H, 28-Me), 0,90 (s, 3H, 28-Me), 0.82 (s, 3H, 20-Me), 0.72 (s, 3H, 19-Me), 0.63 (s, 3H, 30-Me), 1.27 (s, 3H, 26-Me), 1.07 (s, 3H, 27-Me), 3.04,3.00 (dd, J = 6.7,1H, 3-HOH), 3.35,3.31 (m, J = 16.7,1H, 22-CHOH). ^13^C-NMR: see Table 1.

### 3.3. Effects of Compounds on Cell Viability and Apoptosis Rate of B16 Melanoma cells

Fa-a and Fa-b appeared to have some cytotoxic and apoptotic rates, a more highly cytotoxic and apoptotic rates (Fc-a), and a less cytotoxic and apoptotic rates (Fb-a, Fb-a, Fd-a) and also could be showed in Table 2. The results showed that in the cell viability assay, Fa-a and Fa-b did not have appreciable cytotoxic activity at a dose of 100 *μ*M with 11.69%, 4.55%, but reduced viable cells slightly at the higher doses with 34.68%, 29.98%, and 14.97%. Fb-a, Fb-b, and Fd-a had a little or no cytotoxic effect as a whole. Fc-a had highest cytotoxic effect in dose-dependent manner than that of other compounds. Flow cytometry results revealed that the apoptotic rates of B16 melanoma cells with Fc-a (100, 200, and 400 *μ*M) were significantly higher than that of other compounds, with apoptotic rates being 10.12%, 25.44%, and 30.51%. But Dooley [28] previously speculated that a desirable skin-whitening agent should inhibit melanin synthesis in melanosomes by acting specifically to reduce the synthesis or activity of tyrosinase and with little or no cytotoxicity. Hence, Fc-a was not used further due to its greater cytotoxicity on the B16 melanoma cells. Fa-a and Fa-b have certain amount of apoptotic rates of B16 melanoma cells. While Fb-a, Fb-b, and Fd-a had less apoptotic rates (<7% at 400 *μ*M) than that of kojic acid.

### 3.4. Effect of Compounds on the Cellular Tyrosinase Activity and Melanin Content in B16 Melanoma Cells

**Figure 3:**
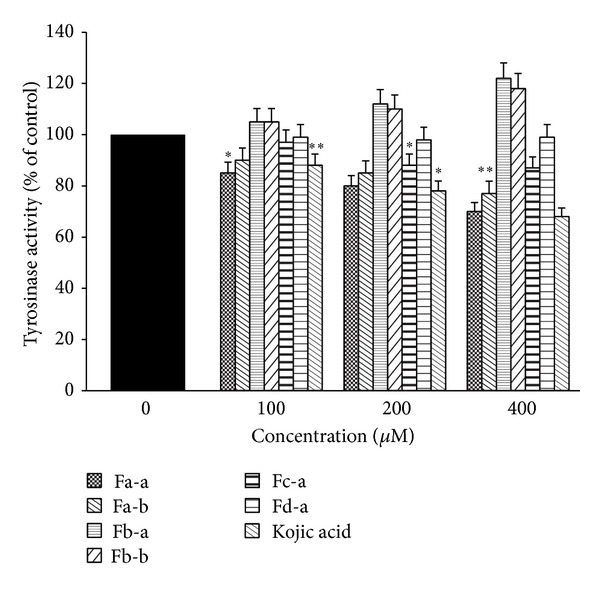
Effects of test compounds and kojic acid on cellular tyrosinase activity in B16 melanoma cells. Data are expressed as a percentage of control which was set at 100%. Each column represents the mean ± SD of three independent experiments. *P < 0.05 and **P < 0.01 compared with the control.

**Figure 4:**
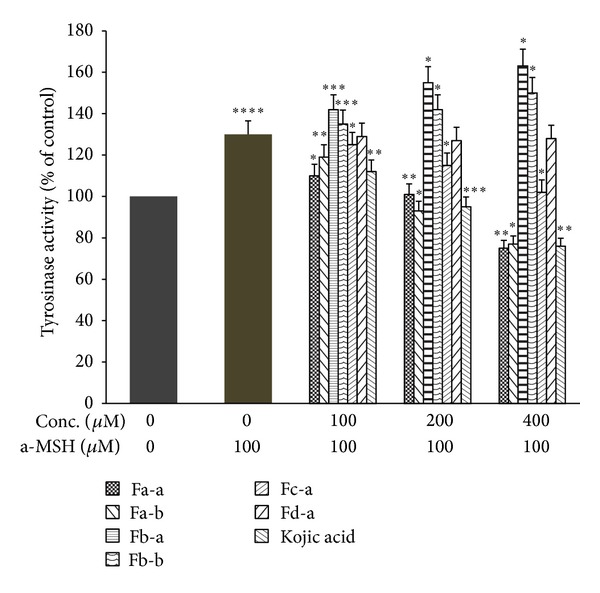
Effects of test compounds and kojic acid on cellular tyrosinase activity in a-MSH-stimulated B16 melanoma cells compared with kojic acid. The cells were incubated with 100 *μ*M a-MSH alone or together with increasing doses of tested compounds or kojic acid for 72 h following which cellular tyrosinase activity was measured. Data are expressed as a percentage of control which was set at 100%. Each column represents the mean ± SD of three independent experiments. ****P < 0.001 versus control group (without a-MSH). ***P < 0.005, **P < 0.01, and *P < 0.05 versus a-MSH-treated group.

**Figure 5:**
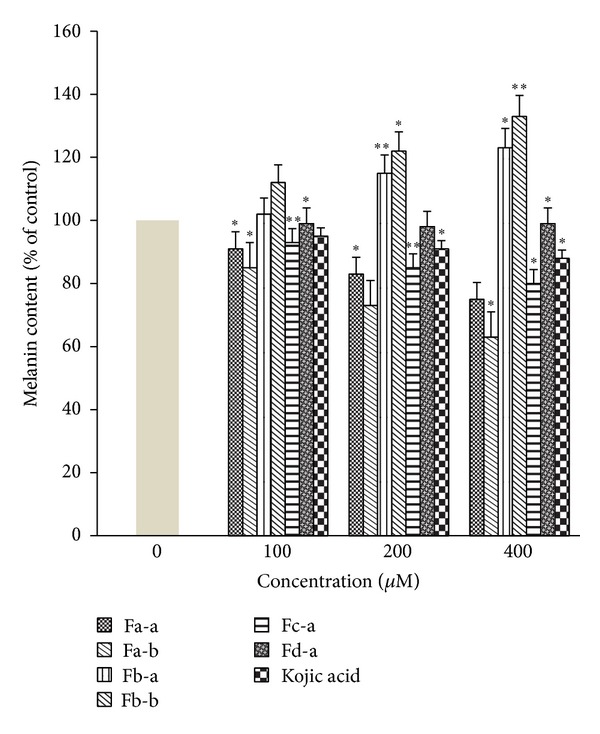
Effects of test compounds and kojic acid on cellular melanin content in B16 melanoma cells. The control readings were set as 100%. Data are expressed as a percentage of control which was set at 100%. Each column represents the mean ± SD of three independent experiments. **P < 0.01, *P < 0.05 compared with the control.

**Figure 6:**
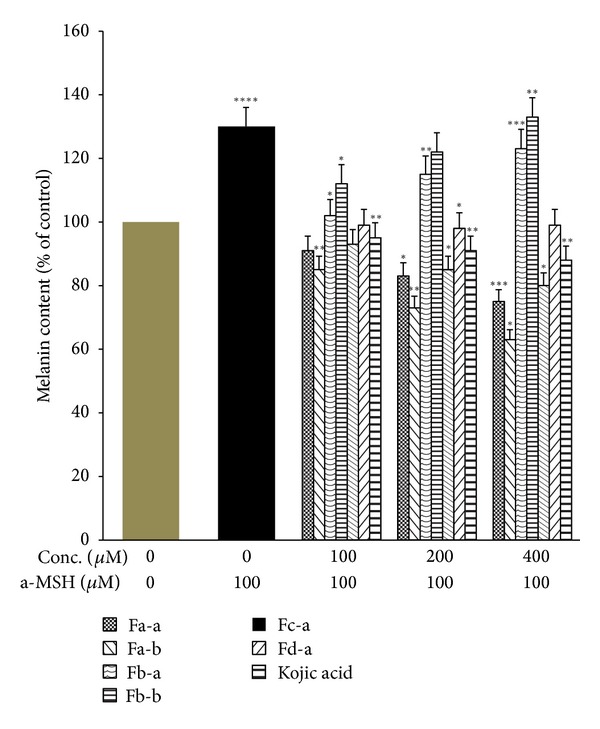
Effects of test compounds and kojic acid on cellular melanin content in a-MSH-stimulated B16 melanoma cells compared with kojic acid. The cells were incubated with 100 *μ*M alone or together with increasing doses of tested compounds or kojic acid for 72 h following which total cellular melanin activity was measured. Baseline melanin content in control wells not exposed to a-MSH and any test compounds or kojic acid was set at 100%. Data from experimental wells were expressed as a percentage of control. Each column represents the mean ± SD of three independent experiments. ****P < 0.0005 versus control group (without a-MSH). ***P < 0.001, **P < 0.01, and *P < 0.05 versus a-MSH-treated group.

The B16 cells line was used because they produce melanin and contain tyrosinase which is associated with melanogenesis under *α*-MSH activation. B16 cells are easy to culture in vitro  [29, 30] and kojic acid as a positive control [17].  Figure 3 demonstrated that Fa-a and Fa-b significantly reduced cellular tyrosinase activity in B16 melanoma cells in the absence of *α*-MSH stimulation in the dose-dependent manner. At 400 *μ*M of Fa-a and Fa-b, they induced significant inhibition on cellular tyrosinase activity by 30.01 and 23.01%, respectively. Fc-a and Fd-a induced slight or no inhibition on cellular tyrosinase activity. On the contrary, Fb-a and Fb-b increased significant cellular tyrosinase activity with 21.24 and 18.21% at 400 *μ*M. Upon exposure to 100 *μ*M *α*-MSH alone, the cellular tyrosinase activity of B16 melanoma cells was significantly increased, compared to the controls (Figure 4). Fa-a and Fa-b were also able to inhibit the increase in cellular tyrosinase activity in a-MSH-stimulated B16 melanoma cells.  Figure 5 showed that Fa-a and Fa-b reduced cellular melanin content in the absence of *α*-MSH stimulation B16 melanoma cells as well as in *α*-MSH-stimulated B16 melanoma cells, compared to *α*-MSH-treated group without compounds in Figure 6. Fb-a and Fb-b had no significant inhibition effect on melanin content as well as cellular tyrosinase activity in B16 melanoma cells. Both compounds had significantly increased melanin content and cellular tyrosinase activity in the presence or absence of 100 *μ*M *α*-MSH. However, due to the slightly cytotoxic effects of Fb-a, Fb-b, they would be used for treatment with vitiligo [31]. Fa-a and Fa-b significantly inhibited cellular tyrosinase activity as well as melanin content in B16 melanoma cells. The decrease in cellular tyrosinase activity could not be attributed to the smaller number of viable cells present because assays were normalised to use the same quantity of protein from each well. Thus the inhibition of tyrosinase activity was credible [21]. The fact that Fa-a and Fa-b were also able to inhibit the increase in cellular tyrosinase in a-MSH-stimulated B16 melanoma cells provides further evidence of the direct action of Fa-a and Fa-b on inhibition of cellular tyrosinase and melanogenesis. However, due to the slightly cytotoxic effects of Fa-a and Fa-b, they would be used for skin whitening. Fd-a did show a slight inhibitory activity as well as cellular tyrosinase activity, and this could be due to a polar agent present in water fraction. However, the inhibitory effect was small and it was not economically feasible to be developed. Kojic acid also reduced tyrosinase activity as well as melanin content in the absence or present of *α*-MSH stimulation in the dose-dependent manner.

### 3.5. Kinetic Analysis of Tyrosinase Activity Inhibition by Compounds

**Figure 7:**
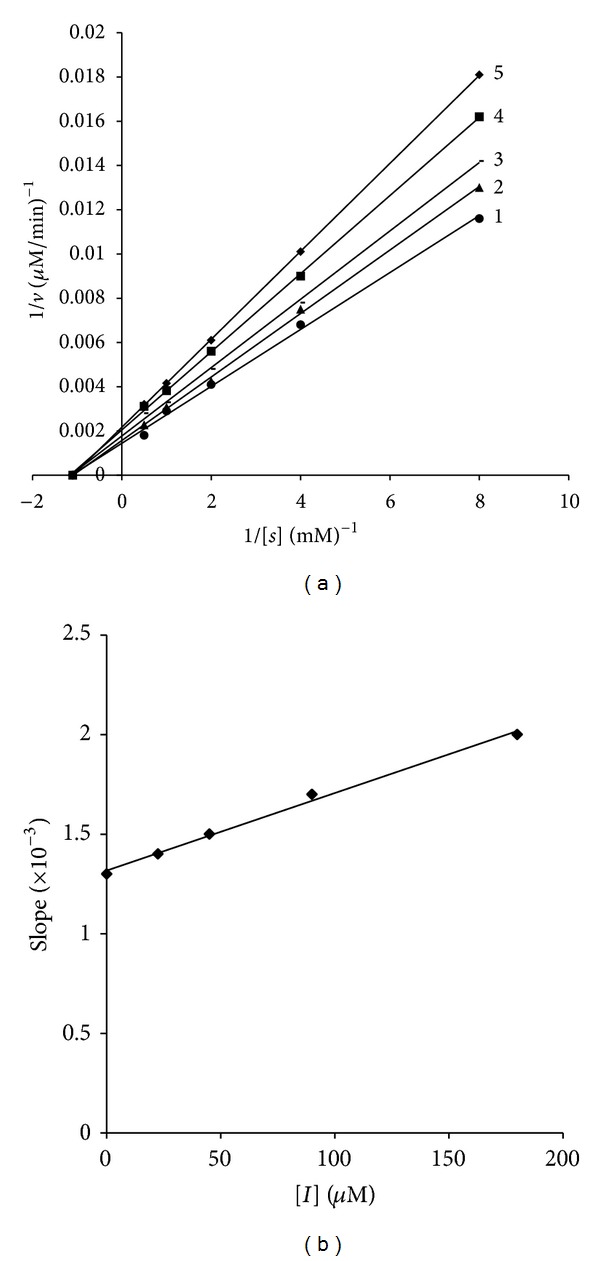
(a) Inhibitory effects of Fa-a on tyrosinase activity in B16 melanoma cells. Lineweaver-Burk plots in the absence (control) or in the presence of Fa-a with L-DOPA as the substrate are shown. Concentrations of Fa-a for the curve 1–5 at 0, 25, 50, 100, and 180 *μ*M, respectively. (b) represents the secondary plot of the slope of the straight lines versus concentration of Fa-a.

**Figure 8:**
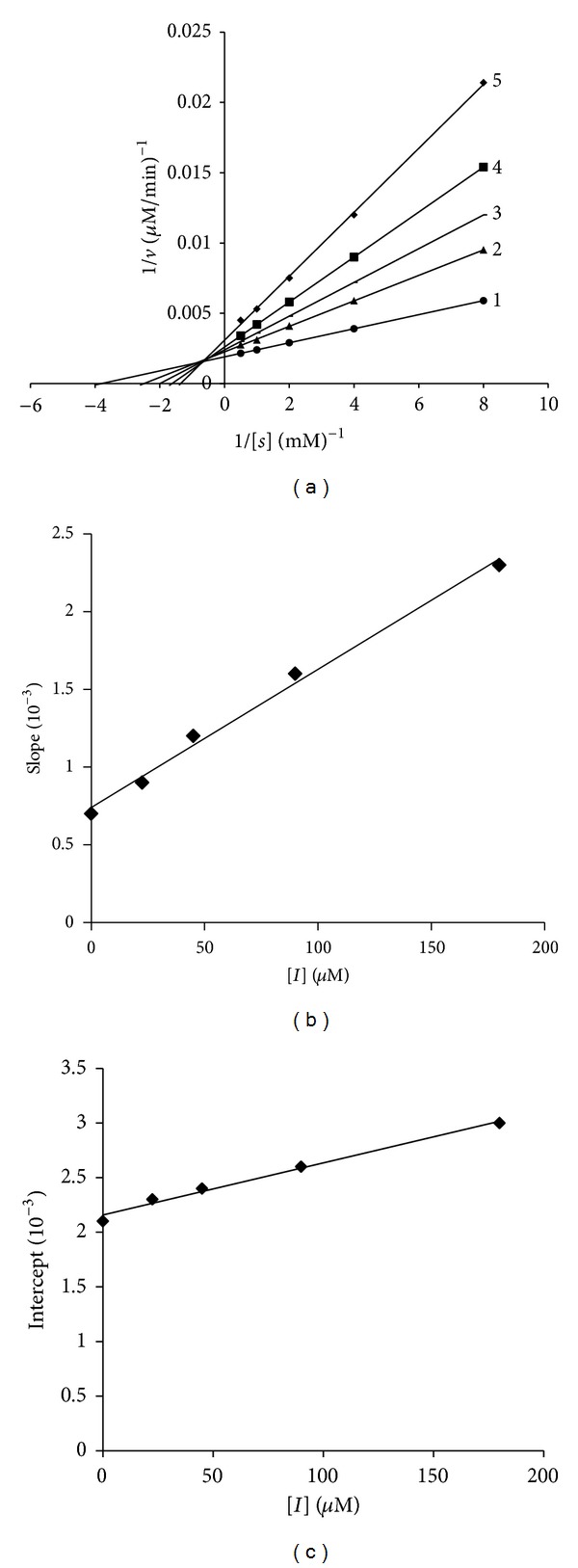
(a) Inhibitory effects of Fa-b on tyrosinase activity in B16 melanoma cells. Lineweaver-Burk plots in the absence (control) or in the presence of Fa-b with L-DOPA as the substrate are shown. Concentrations of Fa-b for the curve 1–5 at 0, 25, 50, 100, and 180 *μ*M, respectively. (b) The plot of slope versus the concentration of Fa-b for determining the inhibition constants *K*_I_. (c) The plot of intercept versus the concentration of Fa-b for determining the inhibition constants *K*_*IS*_.

Fa-a and Fa-b significantly and Fc-a slightly reduced the tyrosinase activity, Fb-a and Fb-b significantly increased the tyrosinase activity of B16 melanoma cells, and Fd-a had a little or no inhibition effect on the tyrosinase activity. So Fa-a and Fa-b were investigated to examine their mechanism of action. We performed an enzyme kinetics study of Fa-a and Fa-b in B16 melanoma cells based tyrosinase assays with various concentrations of the L-DOPA substrate. A Lineweaver-Burk plot of the data was shown in Figures 7 and 8; Fa-a acted as a noncompetitive inhibitor with the plots of 1/[*v*] versus 1/[*S*] gave a family of straight lines with different slopes, which intersected one another in the *x*-axis [12]. Fa-b as mixed inhibitor with the Lineweaver-Burk double reciprocal plots yielded a group of lines that intercept in the second quadrant [26]. *K*_I_ and *K*_*IS*_ values were calculated to be 0.4 and 0.4 *μ*M for Fa-a, 0.9 and 0.5 *μ*M for Fa-b, and 0.6 and 0.8 *μ*M for kojic acid, respectively. Fa-a and Fa-b showed similar inhibition effect on tyrosinase with kojic acid. Fa-a and Fa-b showed tyrosinase inhibitory activity (IC_50_ = 5.13, 7.25 *μ*M). Both compounds showed stronger inhibitory effect than kojic acid (IC_50_ = 6.47 *μ*M). However, Fb-a and Fb-b had shown negative inhibition (acceleration) effect (AC_50_ = 9.74, 8.43 *μ*M). The inhibition could be reduced but not overcome by increasing concentrations of substrate. This reflected an allosteric effect where the compounds bind to different sites on tyrosinase.

## 4. Conclusions

Six compounds isolated from* I. obliquus* were characterized and evaluated for their tyrosinase inhibitory activity in B16 melanoma cells. Among them, Fa-a and Fa-b were potentially the most interesting. They reduced cellular tyrosinase activity and melanin content and displayed a noncompetitive and mixed-type mode of inhibition, respectively. On the contrary, Fb-a and Fb-b increased tyrosinase activities as well as melanin content in B16 melanoma cells. They were potentially the most interesting in treatment with vitiligo.

## Figures and Tables

**Table 1:** ^
l3^C-NMR (300 MHZ, DMSO) spectral data of compounds.

position	^ l3^C-NMR data					
	Fa-a	Fa-b	Fb-a	Fb-b	Fc-a	Fd-a
1	40.9(t)	35.6(t)	35.5(t)	36.1(t)		35.6(t)
2	27.3(t)	28.8(t)	27.8(t)	28.3(t)	154.0(d)	27.8(t)
3	78.1(d)	77.2(d)	78.9(d)	78.9(d)	90.1(s)	79.8(d)
4	39.3(s)	39.0(s)	38.8(s)	39.3(s)	137.4(d)	38.9(s)
5	55.9(d)	50.5(d)	50.42(d)	50.8(d)	124.5(d)	50.4(d)
6	18.8(t)	18.4(t)	19.1(t)	18.6(t)	150.9(d)	19.43(d)
7	34.9(t)	28.0(t)	12.6(t)	26.7(t)	167.0(d)	25.6(t)
8	41.2(d)	134.8(s)	134.6(s)	134.4(s)		134.6(s)
9	50.8(d)	143.7(s)	134.2(s)	134.8(s)		134.2(s)
10	37.6(s)	37.0(s)	37.0(s)	37.4(s)		37.0(s)
11	21.1(t)	21.1(t)	20.9(t)	21.4(t)		21.0(t)
12	25.7(t)	30.6(t)	29.1(t)	31.4(t)		29.7(t)
13	37.5d(d)	44.2(s)	44.8(s)	44.9(s)		44.8(s)
14	43.0(s)	49.4(s)	49.5(s)	50.2(s)		49.4(s)
15	27.6(t)	32.8(t)	31.0(t)	31.0(t)		30.9(t)
16	30.0(t)	27.0(t)	31.0(t)	28.6(t)		30.9(t)
17	48.6(s)	48.1(d)	47.2(d)	50.8(d)		47.4(d)
18	48.4(d)	16.0(q)	15.6(q)	15.7(q)		15.7(q)
19	49.1(d)	19.4(q)	18.3(q)	19.5(q)		19.1(q)
20	151.3(s)	47.3(d)	41.7(d)	36.7(d)		42.9(d)
21	29.7(t)	177.4(s)	12.5(q)	19.1(q)		12.5(q)
22	34.2(t)	27.0(t)	73.3(d)	36.7(t)		74.6(d)
23	28.0(q)	26.1(t)	27.2(t)	25.7(t)		27.2(t)
24	15.3(q)	124.3(d)	121.4(d)	125.2(d)		41.1(t)
25	16.0(q)	131.5(s)	134.9(d)	131.3(d)		70.8(s)
26	16.1(q)	25.7(q)	26.5(q)	26.1(q)		30.0(q)
27	15.3(q)	17.9(q)	18.2(q)	17.9(q)		18.0(q)
28	10.4(t)	28.5(q)	28.0(q)	28.4(q)		15.4(q)
29	109.6(q)	15.5(q)	15.3(q)	15.7(q)		27.9(q)
30	19.1(t)	24.5(q)	24.2(q)	24.2(q)		24.4(q)

**Table 2:** Effects of test compounds and kojic acid on cell viability and apoptosis rate in B16 melanoma cells. Control groups (from wells without test material or kojic acid) were set as 100% for cell viability and set as 0% for apoptosis rate. Experimental groups were expressed as a percentage of controls (mean ± SD).

Compounds	Cell viability			Apoptosis rate		
	100 *μ*M	200 *μ*M	400 *μ*M	100 *μ*M	200 *μ*M	400 *μ*M
Control	100	100	100	0	0	0
Fa-a	88.31 ± 3.21∗	75.13 ± 2.65∗	65.32 ± 4.41	9.01 ± 2.21	12.21 ± 3.03	15.31 ± 2.90
Fa-b	96.45 ± 3.08∗	85.32 ± 4.13∗	70.02 ± 2.05	5.11 ± 4.01	6.51 ± 3.31	10.09 ± 4.31
Fb-a	99.34 ± 1.53∗	99.43 ± 1.41	98.24 ± 1.35	3.91 ± 3.27	5.10 ± 2.61∗	6.07 ± 3.53∗∗
Fb-b	99.98 ± 2.56∗∗	96.26 ± 2.61	94.56 ± 3.03	0.31 ± 4.37∗∗∗	2.62 ± 3.33∗∗∗	5.12 ± 2.35∗
Fc-a	73.53 ± 3.11∗∗	45.31 ± 2.56	40.51 ± 2.12	10.12 ± 1.14	25.44 ± 1.53∗∗∗	30.51 ± 1.11∗∗∗
Fd-a	96.70 ± 1.01	97.12 ± 1.12	85.03 ± 1.51	1.21 ± 2.31	2.15 ± 3.92	7.34 ± 3.28
Kojic acid	92.43 ± 4.41∗∗∗	86.08 ± 2.12∗∗	70.91 ± 3.12∗∗	9.45 ± 1.15	12.36 ± 1.19	15.05 ± 2.21

***P < 0.001, **P < 0.01, and *P < 0.05 compared with the control.

## Abbreviations

DMSO: Dimethyl sulfoxide
L-Tyr: L-tyrosine
L-DOPA: L-3,4-dihydroxyphenylalanine
DAD: Diode array detector
*α*-MSH: *α*-Melanocyte stimulating hormone
PHPLC: Preparative high performance liquid chromatography
HPLC: High performance liquid chromatography
IC_50_: 50% inhibiting concentration
AC⁡_50_: 50% accelerating concentration.
